# Subjective perception of life stress events affects long-term pain: the role of resilience

**DOI:** 10.1186/s40359-022-00765-0

**Published:** 2022-03-04

**Authors:** Natalia Kascakova, Jana Furstova, Radek Trnka, Jozef Hasto, Andrea Madarasova Geckova, Peter Tavel

**Affiliations:** 1 Olomouc University Social Health Institute (OUSHI), Palacky University Olomouc, Univerzitní 22, 771 11 Olomouc, Czech Republic; 2 Psychiatric-Psychotherapeutic Outpatient Clinic, Pro mente sana, Heydukova 27, 811 08 Bratislava, Slovakia; 3 Science and Research Department, Prague College of Psychosocial Studies, Hekrova 805, Prague 4, 149 00 Czech Republic; 4 St. Elizabeth College of Health and Social Work, Palackého 1, 811 02 Bratislava, Slovakia; 5 Slovak Medical University, Limbova 12, 833 03 Bratislava, Slovakia; 6 Department of Health Psychology, Faculty of Medicine, P. J. Safarik University, Trieda SNP 1, 040 11 Kosice, Slovakia

**Keywords:** Childhood trauma, Life stressors with high impact on the last year, Resilience, Moderated mediation model, Long-term pain

## Abstract

**Objective:**

There is evidence that experiencing childhood trauma and life stressors across the lifespan together with lower resilience is associated with chronic pain-related conditions. The aim of this study was to explore the potential mediating role of resilience in the relationship between childhood trauma and long-term pain and to explore a possible moderating role of serious life stressors in the last year.

**Methods:**

The participants, drawn from a representative sample of citizens of the Czech Republic (n = 1800, mean age: 46.6 years, 48.7% male), were asked to report various long-term pain conditions, childhood trauma (Childhood Trauma Questionnaire, CTQ), life stressors (Life Stressor Checklist Revised, LSC-R) and resilience (Brief Resilience Scale, BRS) in a cross-sectional face-to-face study conducted in 2016. A conditional process SEM model of moderated mediation was performed.

**Results:**

The occurrence of life stress events affecting the participant’s last year moderated the relationship between childhood trauma, resilience and health. In the group of participants who experienced at least one life stress event affecting their last year, resilience fully mediated the effect of past childhood trauma on long-term pain. In participants who did not experience life stressors with an impact on the last year, the direct path from childhood trauma to health through resilience lost its significance.

**Conclusion:**

The subjective meaning of stress events on one’s life has an impact on the trajectory between childhood trauma and health and acts as a moderator. Resilience may buffer the negative effect of trauma on later long-term pain.

## Introduction

The experience of being abused or neglected by the closest persons who should be providing care, protection and support can lead to severe neurobiological, psychological and somatic damage in the development of a child [1]. Meta-analytic studies clearly indicate a strong association between experiencing childhood trauma—such as physical, emotional, or sexual abuse and emotional and physical neglect—and worse mental and somatic health in adulthood [2, 3], including chronic or long-term pain [4, 5]. Early traumatized people suffering from long-lasting pain often have stress-induced hyperalgesia [6] and are more prone to pain sensitization and pain chronification, which is often accompanied by pain-related anxiety [7]. The presence of both long-term pain and anxiety in individuals experiencing childhood and adulthood trauma has been found in many cross-sectional studies [8–11]. Patients with chronic pain who have a history of abuse showed greater anxiety and higher catastrophizing [12]. However, some individuals develop into competent, well-adjusted and healthy adults despite the adverse experiences in childhood. The key determinant in effective coping with adversities seems to be resilience [13, 14].

Resilience is defined as the ability to adapt to stress and adversity [15]. Recent theoretical models have conceptualized resilience as a dynamic process characterized as an interaction between “core” resilience (the physiological basis of resilience and personality characteristics), internal resilience (the skills and resources sourced from interpersonal experiences and exposure to adversities) and external resilience (one’s larger socio-ecological context) [16]. Smith et al. [17] have introduced the concept of resilience as an ability to “bounce back” after facing potential stressors, which overlaps with the concept of the “internal” part of resilience. Their approach showed resilience to be a valuable personal resource associated with health-rated measures also when controlling for the other positive characteristics and resources [18]. In contrast with stable personal characteristics (such as optimism as the “core” personal characteristic), this ability to bounce back seems to be more malleable and more easily modified by interventions [18].

The importance of resilience in long-term pain is getting increasing attention. Pain resilience—the ability to maintain positive physical and emotional functioning despite pain [19–21]—can help a person live a meaningful life despite the presence of pain. Resilience has been found to predict lower unpleasantness of pain affect in healthy adults [22] as well as better adjustment to pain and pain acceptance in patients with chronic pain [23]. Karoly and Ruehlman [24] showed that high-resilient individuals with chronic pain had a more adaptive coping style, pain attitudes, health care and medication utilization patterns and weaker catastrophizing tendencies in comparison to low-resilient individuals. High-resilient individuals also reported stronger positive emotions and lower day-to-day pain catastrophizing compared with low-resilient individuals [25].

Interestingly, the role of resilience as a mediator or moderator between childhood trauma and long-term pain does not seem to have been sufficiently explored in population samples. Several recent studies have tested the mediation effect of resilience on subjective physical and mental health in adults [26, 27]. Other studies focusing on young adults and students have shown only a partial mediation effect of resilience [28–30]. The direct and indirect paths between childhood adversities and adulthood health thus need to be further explored, possibly with more complex models incorporating the effects of other factors, such as the influence of life stress events which have high impact on one’s current life. Such strongly influencing events could have a more detrimental effect on later health than events with a lower subjective impact.

Importantly, childhood trauma interacts with the current life stressors in one’s life. The cumulation of life stress events increases the incidence of chronic diseases [31–34]. Moreover, current life stress doubles the effect of childhood abuse on health problems [35]. Some studies have also assessed the subjective emotional perception of negative life stress events on current life and pointed out associations between the subjective impact of some stressor on current life and health [36, 37].

To the best of our knowledge, there is a lack of studies assessing the subjective impact of life stressors in relationship to childhood trauma, resilience and long-term pain in representative samples. Therefore, in addition to exploring the mutual associations between childhood trauma, long-term pain (with or without anxiety) and resilience, our main aim was to explore the possible moderating effect of life stress events with a high impact on one’s life in these complex associations. Considering that resilience can buffer the detrimental effect of childhood trauma on later health, including long-term pain, another aim was to explore the potential mediating role of resilience in the relationship between childhood trauma and long-term pain. Our hypotheses were: (1) long-term pain in adulthood is associated with the occurrence of childhood trauma, and the association is stronger when the anxiety is present; (2) people suffering from long-term pain have a higher occurrence of life stress events with an impact on their last year, and this occurrence is higher when anxiety is present; (3) people suffering from long-term pain have lower resilience, and this association is higher in the presence of anxiety; (4) resilience mediates the link between childhood trauma and long-term pain; (5) life stressors with a subjective high impact on the last year moderate the links between childhood trauma and long-term pain and childhood trauma and resilience.

## Methods

### Sample

The health study was conducted in 2016 on a general population of the Czech Republic. A total of 2184 respondents from the Czech Republic, stratified by gender, age, education and 14 regions, were asked by the administrators to participate in a study on health. The answering rate was 82.4%, 384 of asked respondents refused to engage in the study, mostly men and younger people, due to the length of the questionnaire, non-confidence or reluctance. Ultimately, data from 1800 respondents were collected by trained administrators using face-to-face interviews during September and November 2016. The group of 1800 participants forms a representative sample of the Czech Republic over the age of 15 in regard to gender (48.7% men), age (age 15 to 90, mean age: 46.61), achieved education and regional affiliation. All the participants took part voluntarily; they were not paid, and no other incentives were provided for their participation in the study.

**Fig. 1:**
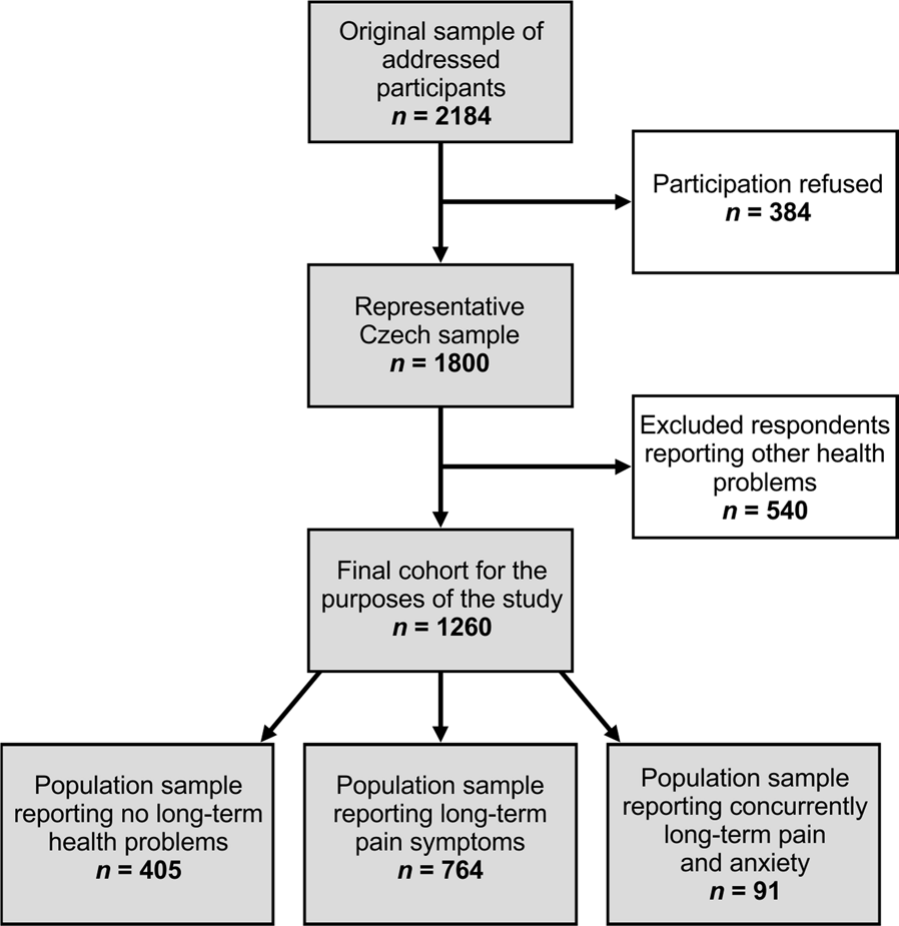
Scheme describing the final sample selection and research groups

Respondents answered a battery of questionnaires regarding early and life-long stress events, attachment, resilience, psychopathology and self-rated health. They were also asked if they suffer from any long-term health problems (e.g. hypertension, allergy, asthma, anxiety or some pain-related condition—such as arthritis, migraine, back pain, pelvic pain or pain of unclear origin). For the purposes of this study, we identified 405 respondents reporting no long-term health problems (the “healthy” community sample), 764 respondents reporting some long-term pain condition (but not reporting anxiety) and 91 respondents reporting both anxiety and some long-term pain condition. An additional 540 respondents reporting other health problems than long-term pain conditions and/or anxiety were excluded. The final research sample thus consisted of 1260 respondents (Fig. 1).

All participants were informed in advance about the main topics of the study, its expected duration (approximately 45–60 min) and procedures, confidentiality and data protection rules, the contact for questions about the research and research participants’ rights, as well as the right to decline to participate and to withdraw from the research once participation began. Respondents agreed with the electronic informed consent, including the data protection declaration, before their participation in the study. Parental informed consent from parents for adolescents ≥ 15 years was obtained prior to the study. The study was conducted at the participants’ homes.

### Measures

#### Sociodemographic data

Participants reported gender (female or male), age (continuous), living arrangement (living with parents or siblings, alone, with partner in a partnership or a marriage) and education (primary school, completed apprenticeship, secondary school graduated and university or college).

#### Long-term health complaints

Long-term health complaints were measured by the item “Do you have some long-lasting disorder or disability? Please, mark all possibilities which are related to you.” Respondents chose from the ensuing list: ischemic heart disease, hypertension, cerebral insult/hemorrhage, allergy, dermatitis (eczema), chronic pulmonary disease, asthma, cancer, diabetes, obesity, gastric and duodenal ulcer, inflammatory bowel disease, arthritis, back pain, migraine, pelvic pain, pain of unclear origin, diseases of the thyroid gland, anxiety, other disease, or no disease. For this study, a long-term pain variable was derived. It included arthritis, back pain, migraine, pelvic pain and pain of unclear origin and was categorized into three values: 1 = no long-term health problems (“healthy”), 2 = long-term pain, 3 = long-term pain and anxiety.

#### Childhood trauma

The Childhood Trauma Questionnaire (CTQ) is a retrospective self-report measuring the severity of five different types of childhood trauma: physical abuse, emotional abuse, sexual abuse, emotional neglect and physical neglect [38]. Each subscale has five items rated on a 5-point Likert-type scale with response options ranging from (1) never true to (5) very often true. The Czech version of the CTQ has been showed to be both reliable and valid [39]. In this study, childhood trauma was considered a latent variable. The CTQ subscales were summed prior to the analyses.

#### Life stressors

The Life Stressors Checklist—Revised (LSC-R) is a 30-item index of lifetime trauma exposure developed especially to include life events that are important stressors [40]. The Czech version of the LSC-R, previously used in a study on the occurrence of stressors in the Czech population and their association with health [41], was used. The advantage of the LSC-R is that, in addition to catching stressors, it measures the personal meaning of stressors and the impact of the stressor on the last year of respondent, coded from 1 (no impact) to 5 (extremely high). For purposes of the analyses, stressors with a high impact (values 4 and 5) were coded as stressors with a high impact on respondents in the last year.

#### Resilience

The Brief Resilience Scale (BRS) was developed by Smith et al. [17] for assessing individuals’ ability to recover or “bounce back” from stressful circumstances. The BRS consists of 6 items assessed on a 5-point Likert scale from 1 (strongly disagree) to 5 (strongly agree). The Czech version of the BRS has shown good psychometric properties, validity and reliability [42]. In this study, resilience was considered a latent variable.

### Statistical analyses

All the statistical computing was performed using the R software 3.6.3 and its packages (R Foundation for Statistical Computing, Vienna, Austria) [43]. The descriptive characteristics of the data were evaluated by means, standard deviations (SD), frequencies and percentages. Our data was not normal (Shapiro–Wilk test, *p* < 0.001 in all the scale variables); thus, the comparison of the groups of respondents was assessed through techniques without the normality assumption: the Kruskal–Wallis test with a Bonferroni correction for multiple group testing was used to compare age, CTQ score and BRS between groups, and Pearson’s χ^2^ test was used to compare gender and occurrence of life stress events affecting the person’s life in the past year. Structural equation models (SEM) were used to assess the mediating and moderating relationship between childhood trauma, resilience, chronic pain and life stress. For fitting the SEM models, the R Lavaan package was used [44]. For estimating the parameters, the diagonally weighted least squares method (DWLS) based on polychoric correlations was used. Several model fit indices were evaluated: the comparative fit index (CFI) > 0.95, the Tucker–Lewis index (TLI) > 0.95, the root mean square error of approximation (RMSEA) < 0.08 and the standardized root mean square residual (SRMR) < 0.08 were considered a good fit [45].

**Fig. 2:**
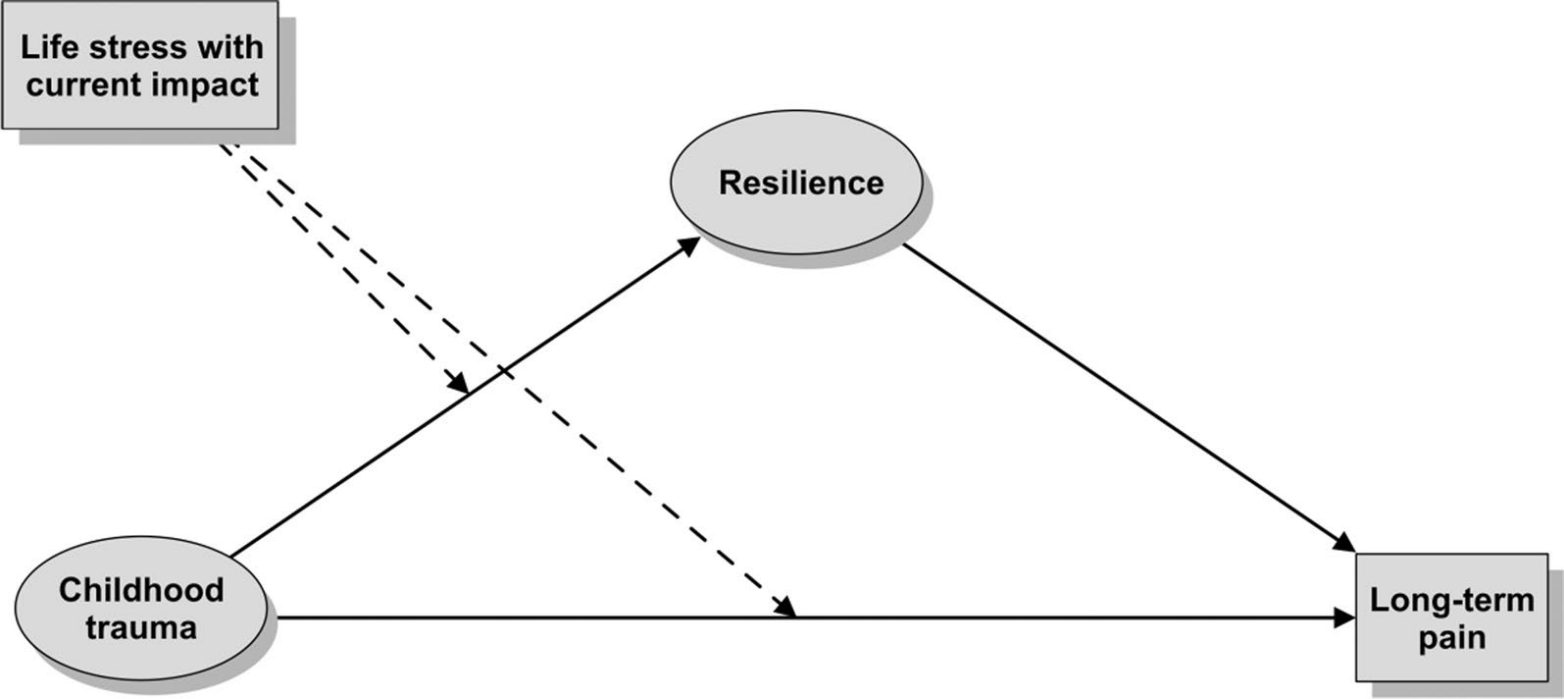
Conceptual representation of the conditional process model, i.e. moderated mediation

In the mediation analysis, childhood trauma and resilience were modeled as latent variables. Childhood trauma was measured by the subscales of the Childhood Trauma Questionnaire (CTQ). Resilience was measured by the items of the BRS scale. The mediation effect was tested in the Lavaan package with bootstrap standard errors. The number of bootstrap draws was 5000. A conditional process model of moderated mediation discussed in Hayes and Rockwood [46] was then fit to assess the effect of childhood trauma on chronic pain mediated by resilience and moderated by life stress events affecting the respondent’s life in the past year (see Fig. 2). To assess the moderating effect of life stress events, multiple group analysis (MGA) of SEM models was used. The unconstrained and constrained models were compared using the R semTools package [47]. The significance level was set at *p* < 0.05 for all statistical significance testing.

## Results

### Characteristics of the sample

The descriptive characteristics of the sample are presented in Table 1. The sample comprised participants reporting no long-term health problems (32.1%), those suffering from long-term pain (60.6%) and those suffering from long-term pain with anxiety (7.2%). The mean age of the sample was 46 years; 52.6% were women. The proportion of women was significantly higher in the groups reporting long-term pain with and without anxiety symptoms than in the group without any long-term health problems (χ^2^(2) = 33.68, *p* < 0.001, Cohen’s d = 0.33). Participants reporting long-term pain with and without anxiety symptoms were significantly older (H(2) = 204.56, *p* < 0.001, Cohen’s d = 0.88), reported a higher level of emotional neglect and abuse (H(2) = 24.31, *p* < 0.001, Cohen’s d = 0.27) and physical neglect during childhood (H(2) = 29.70, *p* < 0.001, Cohen’s d = 0.30) and showed significantly lower resilience (H(2) = 94.83, *p* < 0.001, Cohen’s d = 0.57) than those reporting no long-term health problems; see Table 1. Participants reporting no long-term health problems also reported lower occurrence of life stress events affecting their life in the past year (χ^2^(2) = 91.05, *p* < 0.001, Cohen’s d = 0.56). In participants reporting long-term pain with anxiety the proportion of women was significantly higher (χ^2^(1) = 6.93, *p* = 0.009, Cohen’s d = 0.18), and there was a higher occurrence of emotional and physical neglect (H(2) = 24.31, *p* < 0.001, Cohen’s d = 0.27, and H(2) = 29.70, *p* < 0.001, Cohen’s d = 0.30, respectively), lower resilience (H(2) = 94.83, *p* < 0.001, Cohen’s d = 0.57) and a higher proportion of life stress events affecting their last year (χ^2^(1) = 22.51, *p* < 0.001, Cohen’s d = 0.33) compared to those reporting long-term pain without anxiety. The prevalence of the types and number of long-term pain conditions in relationship to life stressors and resilience is presented in Table 2. Respondents who reported more long-term pain symptoms had a lower level of resilience, and a higher proportion of them experienced stressful life events affecting their life in the past year.

**Table 1:** Descriptive characteristics of the sample

Characteristics	A. No long-term health problems	B. Long-term pain	B versus A	C. Long-term pain with anxiety	C versus A	C versus B
	N = 405	N = 764	*P* value	N = 91	*P* value	*P* value
Age: Mean (SD)	36.4 (14.3)	51.2 (16.3)	< 0.001	51.6 (18.6)	< 0.001	n.s
Gender: N (%)						
Male	235 (58.0)	335 (43.8)	< 0.001	27 (29.7)	< 0.001	0.009
Female	170 (42.0)	429 (56.2)		64 (70.3)		
CTQ: Mean (SD)						
Emotional abuse (EA)	6.50 (2.23)	7.20 (3.07)	0.003	7.96 (3.42)	< 0.001	n.s
Physical abuse (PA)	5.63 (1.88)	6.01 (2.31)	0.009	5.74 (2.08)	n.s	n.s
Sexual abuse (SA)	5.42 (1.58)	5.50 (1.82)	n.s	5.68 (2.13)	n.s	n.s
Emotional neglect (EN)	9.86 (4.45)	10.59 (4.56)	0.012	12.59 (5.29)	< 0.001	0.002
Physical neglect (PN)	6.91 (2.64)	7.41 (2.75)	0.001	8.41 (2.95)	< 0.001	0.005
BRS: Mean (SD)	3.22 (0.70)	2.96 (0.68)	< 0.001	2.43 (0.68)	< 0.001	< 0.001
Occurrence of life stress events affecting the respondents’ life in the past year: N (%)						
Yes	45 (11.1)	224 (29.3)	< 0.001	49 (53.9)	< 0.001	< 0.001
No	360 (88.9)	540 (70.7)		42 (46.1)		

*P* values correspond to the χ^2^ and Kruskal–Wallis tests; n.s. = non-significant (*p* > 0.05)

**Table 2:** Prevalence of various types of long-term pain and number of pain symptoms in relationship to resilience and reported life stressors affecting the person’s life in the past year

Research groups	Resilience		Differences between groups	Life stress events affecting the respondents’ life in the past year			
				No		Yes	
	n	Mean (SD)		n	%	n	%
Number of pain symptoms							
A. No long-term problems (healthy)	405	3.22 (0.69)	A–B***, A–C***, A–D***, B–D*	360	88.9	45	11.1
B. 1 long-term pain symptom	616	2.96 (0.69)		435	70.6	181	29.4
C. 2 long-term pain symptoms	197	2.79 (0.70)		132	67.0	65	33.0
D. ≥ 3 long-term pain symptoms	42	2.67 (0.79)		15	35.7	27	64.3
Type of long-term pain							
Arthritis	121	2.82 (0.64)		82	67.8	39	32.2
Backpain	631	2.91 (0.70)		422	66.9	209	33.1
Migraine	223	2.87 (0.75)		148	66.4	75	33.6
Pelvic pain	68	2.71 (0.71)		34	50.0	34	50.0
Pain of unclear origin	99	2.72 (0.73)		61	61.6	38	38.4

*p < 0.05, **p < 0.01, ***p < 0.001, according to the Kruskal–Wallis test

### Testing the mediating effect of resilience

To test the mediating effect of resilience on the relationship between childhood trauma and long-term pain, SEM models were employed. The studied SEM model showed acceptable values for the CFI and TLI indices and for RMSEA and SRMR (Table 3).

**Table 3:** The parameters and fit indices of the SEM model used in the mediation analysis

Path	Standardized parameter estimate	Standard error	P-value	CFI	TLI	RMSEA (90% CI)	SRMR
CTQ → long-term pain	0.131	0.034	< 0.001	0.973	0.965	0.053 (0.046‒0.060)	0.053
CTQ → BRS	− 0.105	0.031	0.001				
BRS → long-term pain	− 0.333	0.032	< 0.001				

*CTQ* Childhood Trauma Questionnaire, *BRS* Brief Resilience Scale

**Fig. 3:**
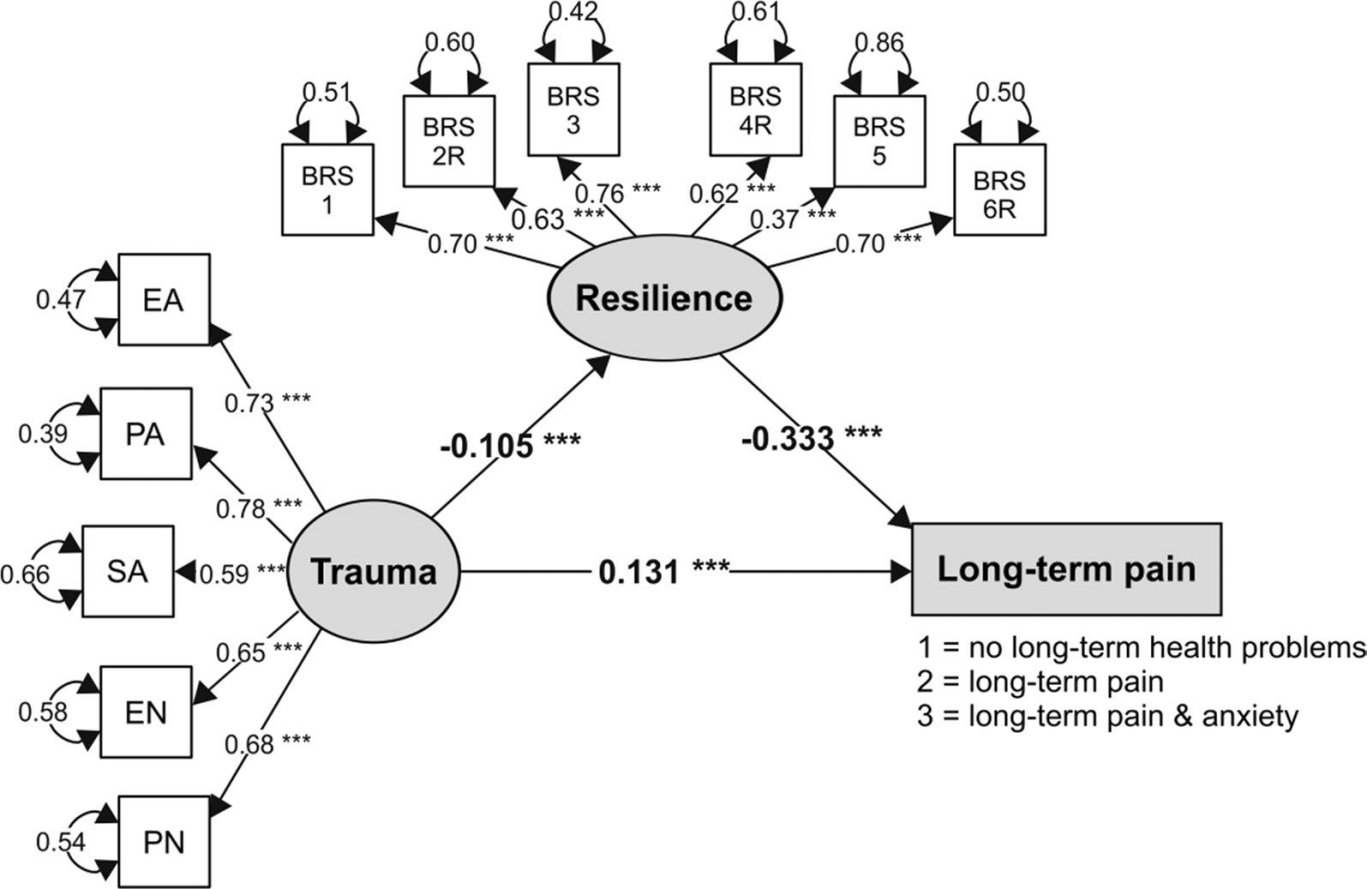
The mediation SEM model of childhood trauma (CTQ), resilience (BRS) and long-term pain. *Note*. ***p < 0.001. EA = Emotional abuse, PA = Physical abuse, SA = Sexual abuse, EN = Emotional neglect, PN = Physical neglect. All coefficients are standardized.

As shown in Fig. 3 and Table 3, a higher level of childhood trauma had a significant direct effect on long-term pain and on resilience. Specifically, a higher level of childhood trauma increased the likelihood of long-term pain and decreased the level of participants’ resilience. Resilience had a significant direct effect on long-term pain: lower resilience increased the likelihood of long-term pain of participants. Both the direct and indirect paths from childhood trauma to long-term pain remained statistically significant; thus, only a partial mediation effect of resilience was found in our data (standardized indirect effect = 0.02, SE = 0.005, *p* = 0.001; standardized total effect = 0.08, SE = 0.016, *p* < 0.001).

### Moderated mediation model

Since the resilience did not fully mediate the relationship between childhood trauma and long-term pain, a somewhat more complicated model was considered: the mediation model of childhood trauma, resilience and long-term pain was assumed to be moderated by the occurrence of life stress events affecting the participant’s life in the past year. A conditional process model according to Hayes and Rockwood [46] was used. Such a process is often called moderated mediation.

**Fig. 4:**
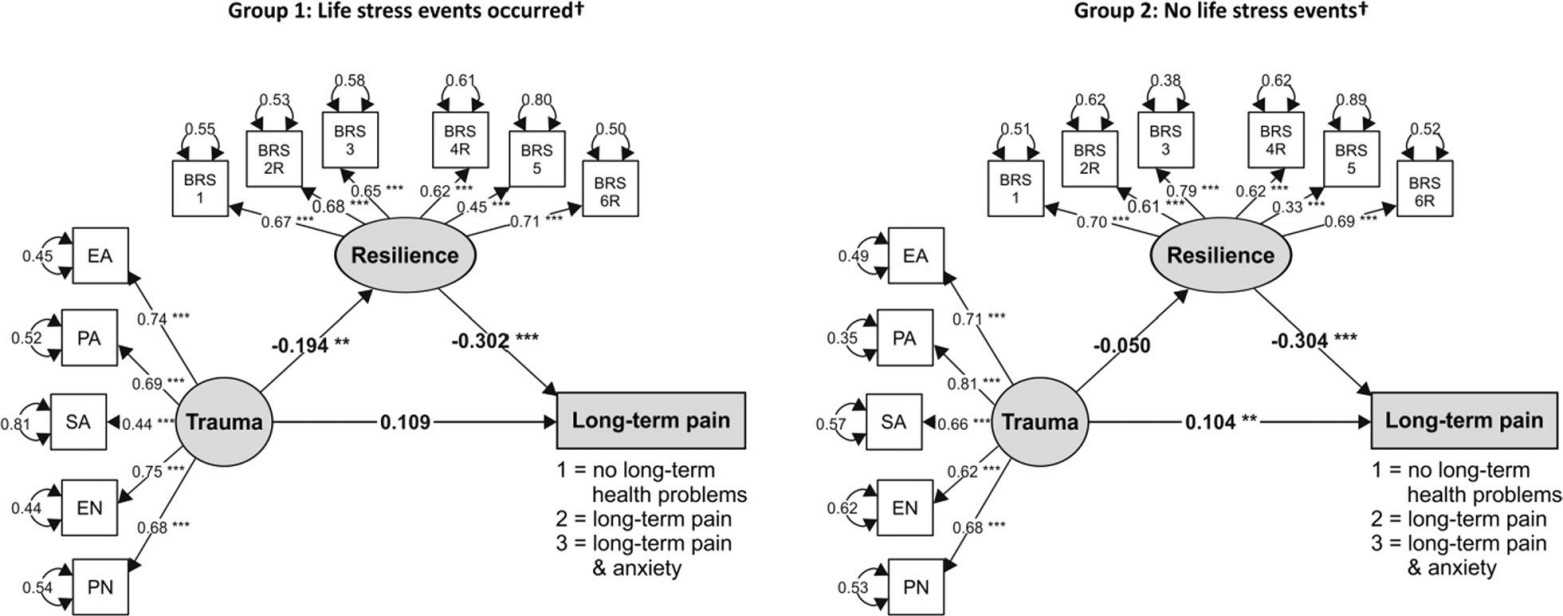
The SEM models of the mediating effect of resilience (BRS) on the relationship between childhood trauma (CTQ) and long-term pain, moderated by life stress events affecting the respondents’ life in the past year (LSC-R). *Note* ***p < 0.001, **p < 0.01. EA = Emotional abuse, PA = Physical abuse, SA = Sexual abuse, EN = Emotional neglect, PN = Physical neglect. †Life stress events affecting the respondents’ life in the past year. All coefficients are standardized

First, the sample was divided into a group of participants who experienced at least one life stress event affecting their life in the past year (LSPY) (n = 318) and a group that did not experience LSPY (n = 942). The mediation model was then fit to the data in both groups. As depicted in Fig. 4, in the group that experienced at least one LSPY, resilience fully mediates the effect of childhood trauma on long-term pain. In this group, a higher level of childhood trauma significantly decreased the level of resilience (β = − 0.19, SE = 0.06, *p* = 0.002), and lower resilience significantly increased the likelihood of long-term pain (β = − 0.30, SE = 0.07, *p* < 0.001). On the other hand, in the group that did not experience LSPY the indirect path from childhood trauma to chronic pain through resilience loses its significance. Therefore, LSPY clearly affects the relationship between childhood trauma, resilience and long-term pain.

To test the moderating effect of the LSPY, a multiple group analysis (MGA) of SEM models was used. The unconstrained (baseline) and constrained models were compared. Freeing the constraints in the model resulted in a significant improvement in the fit (freeing intercepts yielded ∆χ^2^(10) = 77.73, *p* < 0.001; freeing residual variances yielded ∆χ^2^(2) = 47.85, *p* < 0.001). We can thus conclude that LSPY moderates the relationship between childhood trauma, resilience and long-term pain. Figure 4 and Table 4 show the SEM results for the association between childhood trauma, resilience and long-term pain for people experiencing LSPY and those who did not experience LSPY.

**Table 4:** The parameters and fit indices of the SEM models used in the moderated mediation analysis

Path	Standardized parameter estimate	Standard error	*p* value	CFI	TLI	RMSEA (90% CI)	SRMR
Group 1: Life stress events occurred†							
CTQ → long-term pain	0.109	0.070	0.118	0.989	0.985	0.031 (0.001‒0.050)	0.055
CTQ → BRS	− 0.194	0.064	0.002				
BRS → long-term pain	− 0.302	0.071	< 0.001				
Group 2: No life stress events†							
CTQ → long-term pain	0.104	0.039	0.007	0.966	0.956	0.059 (0.051‒0.067)	0.060
CTQ → BRS	− 0.050	0.034	0.133				
BRS → long-term pain	− 0.304	0.037	< 0.001				

*CTQ* Childhood Trauma Questionnaire, *BRS* Brief Resilience Scale

^†^Life stress events affecting the respondents’ life in the past year

## Discussion

This study on a representative sample revealed associations between childhood trauma, resilience and long-term pain. Participants reporting long-term pain conditions with or without anxiety reported significantly higher occurrence of life stress events strongly affecting their life in the past year compared to people reporting no long-term health problems. Resilience partly mediated the relationship between childhood trauma and long-term pain. The moderated mediation showed that the occurrence of life stress events with a high effect on the participant’s last year moderates the relationship between childhood trauma, resilience and long-term pain. In the group of participants who experienced at least one life stress event with a high effect on their last year, resilience fully mediates the effect of childhood trauma on the presence of long-term pain. On the other hand, in participants who did not experience life stressors with a high impact on the last year, the direct path from childhood trauma to long-term pain through resilience lost its significance.

Participants reporting long-term pain with and without anxiety symptoms reported a higher level of emotional neglect and abuse as well as physical neglect during childhood, which is in line with a recent German population study [5] that revealed associations between childhood trauma and long-lasting pain symptoms. The presence of long-term pain (with or without anxiety) was associated with a higher occurrence of abuse and neglect in childhood, in line with many cross-sectional studies [8–10] and, moreover, in respondents with long-term pain and anxiety the occurrence of emotional and physical neglect was higher than in respondents with long-term pain without anxiety.

The direct effect of childhood trauma on lower resilience is also in accordance with other empirical evidence in this field [48–51]. Moreover, a recent study with 40 young people [51] showed a dose–response relationship: those with higher scores of adverse events showed lower resilience and more psychopathology.

Participants from the present study reporting long-term pain symptoms reported a significantly higher occurrence of life stress events strongly affecting their life in the past year, and this occurrence was even higher in the group reporting long-term pain with anxiety. The associations between subjective impact of life stressors and the occurrence of long-term pain is a relatively less explored area; nevertheless, the relationship between life stressors—namely as cumulative life stress or a combination of childhood trauma and adulthood life stress, such as interpersonal violence—and medical, long-term or chronic pain symptoms is well explored [31, 32, 34]. A potentially bidirectional relationship between long-term pain and stress should be also considered: experience of long-term pain is a stressor itself and can act as a chronic stress [19]. Although chronic stress and chronic pain are different phenomena, they do overlap; both challenge the body’s homeostasis and both can lead to compromised well-being [52]. Yeung, Arewasikporn and Zautra [21] reviewed a set of stable and modifiable factors in the intra- and interpersonal domains that may foster and/or hinder resilient functioning in chronic pain. They underscore the importance of incorporating social resilience into the development of interventions promoting adaptive functioning in patients with chronic pain.

Our hypothesis, that resilience mediates the relationship between childhood trauma and long-term pain, was not fully supported and the mediation was only partial. In other words, the relationship between childhood trauma and long-term pain still remained significant. In the group of participants who experienced at least one life stress event with a high impact on their last year, resilience fully mediated the effect of childhood trauma on the presence of long-term pain. This is in line with the findings of Karatzias et al. [27] in a population-based study where resilience served as a mediator between multiple potentially traumatic life events and physical and mental health. Faircloth [29], in her thesis on college students, found that resilience partially mediated the relationship between negative life events and well-being. Fischer et al. [30] proposed a multi-dimensional stress structural equation model (SEM) in functional somatic syndromes (FSS) in a sample of 3054 students and found that resilience indirectly lowered the probability of FSS in the direct pathways between childhood trauma, stress reactivity, chronic stress and FSS. Moreover, a German study from a representative sample highlighted that subjects with high resilience showed less distress and somatoform symptoms despite reported childhood adversities [53]. This finding supports the relevancy of suggestions in this field that strengthening the modifiable factors of resilience could lead to better adaptation to long-term or chronic pain conditions, even if patients reported childhood trauma in their anamneses.

Our last hypothesis was fully supported by the results: the occurrence of life stress events with a high impact on the participant’s last year moderated the relationship between childhood trauma, resilience and long-term pain. The subjective emotional perception of negative life events seems to be an important factor in the resulting effect of the event on one’s life. This finding is supported by a German population-based study: a lower present impact of past negative life events was associated with better subjective health [36]. The way that people subjectively assess negative stress events in the longer-term perspective depends on the characteristics of the event (e.g. the time, duration, repeating) and on the individual’s personal protective resources [54], including genetic and epigenetic factors [55]. According to some studies, people with dispositional optimism are less affected by negative stress events [56, 57]. Moreover, high levels of extraversion, openness and conscientiousness and a lower level of neuroticism are associated with less stressor-related affect [58]. On the other hand, optimistic students showed in the case of accumulated negative life stressors worse psychological adjustment than pessimistic students [59]. Probably, people with unrealistic optimism who believe that “things do not go wrong” may be particularly vulnerable when things do go wrong [60]. On the other hand, optimists have more social ties, are more satisfied with their relationships and report greater social support [61]; thus, they have more resources available in their social network in the case of some negative life stress event and may be more prone to seek and accept help.

We propose that social support (as an external resilience resource) within the time of experiencing a stressor can substantially lower the subjective meaning of the perceived negative stress event and attenuate the negative impact on health. In women experiencing partner violence, higher social support was associated with a significantly reduced risk of poor mental and physical health, symptoms of posttraumatic stress disorder and suicide attempts [62]. A study involving 64 women with cancer revealed that only interpersonal loss (and not the loss of financial or work resources) mediated the relationship between earlier interpersonal trauma and current posttraumatic stress disorder and depressive mood [63]. Developmental studies in particular have shown that proximity to a caregiver or to some trustful person is an important modulator of a child’s sense of safety when facing trauma [64]. In the presence of chronic pain, perceived social support, independently with pain coping, was shown to be a predictor of psychosocial adjustment [65]. The exploring of the therapeutic potential of social support in patients with long-term pain and a history of childhood trauma could be a prospective area for future research.

This study has also practical implications. Apart from enhancing external resilience by promoting social support, the results of this research draw attention to therapeutic possibilities for enhancing individual resilience as an effective inner source for facing potential stressors. Although some attributes of resilience are biologically determined [55, 66] resilience skills can be fostered and improved [67]. Relaxation techniques, such as autogenic training, guided imagery, progressive muscle relaxation, hypnosis, etc., are useful tools for enhancing relaxation through downregulation of the sympathetic nervous system involved in stress response [68]. A mindfulness-based stress reduction program can lead to better distress tolerance through enhancing the participant’s mindfulness and resilience [69]. Haase et al. [70] suggested that there is a link between resilience and interception. People who are less aware of the possibility of internal bodily changes are more susceptible to stress and less able to cope with stressors, such as (chronic) pain. These findings indicated that bodily awareness training could be a suitable intervention tool for enhancing resilience in patients suffering from long-term or chronic pain [71]. Further investigation of the relationships between childhood trauma, subjective perception and the impact of life stress on current life, health and resilience is needed.

This study was not focused on post-traumatic stress disorder (PTSD) in relation to long-term pain. The impact of life stress events on current life could be mediated by traumatic memories in the presence of PTSD. Exploration of associations between the subjective impact of life stress on one’s life and PTSD could be valuable for targeted diagnostic and treatment in patients with long-term pain and a history of childhood trauma and lifelong stress events.

### Strengths and limitations

The strength of this study is that it is based on a representative sample. A community sample brings the advantage of examining the link between life stressors, resilience and long-term pain complaints in the whole population. This approach might offer a better overview of the situation than studies based only on patient data from medical facilities.

One limitation is that long-term pain was based on self-report of a diagnosis, and this could be confused with other diagnoses, e.g. migraine could be confused with tension headache. This study did not explore in detail the character of the long-term pain, e.g. its duration, its severity or the nature of the pain. We do not know if the pain endured longer than 3 months, which means that we could not label the pain as “chronic” [72]. On the other hand, the question of whether the participants suffer from some long-lasting health condition implies that when they refer to pain, it would not be acute pain but pain that is present over a longer period of time. The results of national studies suggest that the assessment of long-term pain conditions by self-reports is a valid option in research [73].

A second limitation is that the associations in the models were analyzed through a cross-sectional design, which inhibits causal interpretations. However, in some participants with long-term pain it can be assumed that long-term pain as a stressor could also decrease resilience, and the relationship between resilience and long-term pain in our study may be bidirectional.

A third limitation is that a history of childhood trauma was recalled and reported retrospectively and therefore can be biased. A review study in this field has shown a trend towards the under-reporting of child abuse and neglect when asking respondents in adulthood [74]. Moreover, the face-to-face interview took place at each participant’s home and in adolescent respondents aged ≥ 15 in particular the presence of parents/guardians could have affected the answering, even if they were in another room. On the other hand, the interviewer was an unknown, neutral person for the participant, and prior to the interview the interviewer informed the respondent about confidentiality and data protection rules, which could have increased the likelihood of answering honestly.

Fourthly, age and gender were not assessed as potential confounders in this study. We also anticipated that after including age and gender into the analyses, the effect of childhood trauma on long-lasting pain would be significant, in line with other studies based on representative samples [5, 9].

Finally, the Life Stressor Checklist—Revised which allowed assessing the degree of the impact on one’s current life also contains questions related to child abuse and neglect, which means the variable “life stress events affecting the last year” contains negative events across the whole life span of the respondent. We did not differentiate the time when the life stress event was experienced.

## Conclusion

This study on a representative sample revealed associations between childhood trauma, long-term pain and resilience. The subjective meaning of stress events on one’s life has an impact on the trajectory between childhood trauma and long-term pain and acts as a moderator. Resilience may buffer the negative effect of trauma on later long-term pain. Psychosocial and therapeutic interventions aimed at strengthening resilience could be helpful in buffering the negative effect of childhood trauma and later life stressors on health.

## Data Availability

The datasets generated and analysed during the current study are not publicly available due to the Czech legislation but are available from the corresponding author on reasonable request.
